# Reticulocytosis in screen-printing workers exposed to 2-butoxyethanol and 2-ethoxyethanol

**DOI:** 10.1186/s40557-017-0210-z

**Published:** 2017-11-07

**Authors:** Seng-Ho Song, Seong-Kyu Kang, Won-Jun Choi, Kyeong Min Kwak, Dong-Hoon Lee, Dyuk-Yoon Kang, Sang-Ha Lee

**Affiliations:** Department of Occupational and Environmental Medicine, Gachon University Gil Medical Center, 21, Namdongdaero774 beon-gil, Nadong-gu, Incheon, 21565 Republic of Korea

**Keywords:** 2-Butoxyethanol, 2-Ethoxyethanol, Ethylene glycol ethers, Reticulocytosis

## Abstract

**Background:**

Studies on the hematologic toxicity of ethylene glycol ethers in humans are limited. Therefore, the aim of this study was to examine the association between exposure to solvents (containing 2-butoxyethanol and 2-ethoxyethanol) and hematological effects.

**Methods:**

Thirty-four screen-printing workers who were exposed to 2-butoxyethanol and 2-ethoxyethanol and 37 non-exposed clerical workers were selected using data from the health care facilities that provided regular health screening services. Student’s t-tests and Pearson’s chi-square tests were used to compare differences in hematological parameters between the exposed and the control groups. A multivariate analysis was performed using the multiple logistic regression models to adjust for other variables.

**Results:**

The chi-square test showed the reticulocyte percentages and corrected reticulocyte counts to be significantly higher in the exposed group. The t-tests showed a significant increase in white blood cell counts, reticulocyte percentages, and corrected reticulocyte count (i.e., reticulocyte index) in the exposed group, with *p*-values of 0.002, 0.004, and 0.002, respectively. Multivariate analysis showed the odds ratio for the corrected reticulocyte counts to be 16.30 for the exposed group, when compared with that of the control group.

**Conclusions:**

Exposure to 2-butoxyethanol and 2-ethoxyethanol was significantly associated with reticulocytosis, necessitating the implementation of preventive measures for workers prone to occupational exposure to ethylene glycol ethers.

## Background

Ethylene glycol ethers, especially 2-ethoxyethanol (EE) (CAS No. 110–80-5) and 2-butoxyethanol (BE) (CAS No. 111–76-2), are colorless liquids. They have an amphiphilic structure, with the hydrophilic part interacting with water, and the lipophilic part interacting with organic solvents, which explains their solubility in both water and organic solvents such as oils. Because of this characteristic, EE and BE are widely used in industry as ingredients of paints, inks, water-based cleaners, adhesives, detergents, cosmetics, cutting fluids, agrochemicals, jet fuels, and hydraulic brake fluids [1]. Animal and human studies have shown that EE and BE cause adverse hematological effects through inhalation, dermal absorption, and ingestion. These ethers are oxidized by NAD-dependent dehydrogenases in the liver, testicles, and skin to their corresponding alkoxyacetic acids (AAs), which have been identified as the primary urinary metabolites in humans and animals [2, 3]. AAs are responsible for hematological toxic effects in humans and animals [4–8]. The relationship between ethylene glycol ethers and hematologic toxicity was identified in several animal studies [7–11], but only a few studies in humans exist. A previous study described a genetic polymorphism affecting transcriptional regulation of the human cytochrome P450 2E1 gene, and this polymorphism could influence ether metabolism [12]. The toxicity of ethylene glycol ethers is also known to be species-specific with high toxicity in rodents and low toxicity in humans. Thus, extrapolating the results of animal experiments to humans can lead to overestimation of toxicity [13]. In addition, there are limited epidemiological studies indicating that exposure to BE and EE in humans might account for the hematological effects [4, 5, 8, 14]. It is difficult to clarify any association because of the complicated mixtures of exposed chemicals, including glycol ethers as well as other hematological toxicants, and the existence of limited human exposure data.

We conducted a cross-sectional study of screen-printing workers with occupational exposure to solvents containing BE and EE and office workers without known occupational exposure such as mixed solvents. The objective of the study was to evaluate hematological effects in screen-printing workers chronically exposed to solvents containing BE and EE.

## Methods

### Subjects

The exposure group was all 34 workers belonging to the screen-printing department at a small-sized printing firm working with fewer than 60 workers at Namdong Industrial Complex, Inchon, South Korea. They worked with dye mixing, printing, drying, and product inspection. All the workers had varied working schedules depending on circumstances, such as order quantity or work-type. Nevertheless, the exposure group usually worked 8 h a day for 5 days a week. The exposure group had the worker’s health check-up on December 5th to 7th, 2016. The exposure group included 10 foreign workers, but nationality could not be identified because no data was available. The control group samples were selected from workers who had blood examinations on November 1st to December 31st at the same health care facilities that provided regular health screening services (*n* = 1858). After inclusion of only those aged ≤50 years (*n* = 386) and individuals with no known occupational risk of solvent exposure (e.g., clerical workers including bank clerks, teachers, salespersons, and other officers), 37 clerical workers remained in the control group, and there were no foreigners.

### Job description and manufacturing processes

Workers in the exposed group were involved in labeling or text printing on the front glass of cell phones. Regular operations in the screen-printing process included dye mixing, printing, and drying. The dye mixing process involved diluting a dye with a solvent in a ratio of dye to solvent with variations of approximately 30 to 70%. The next process was printing. A silk-film board was placed on the product, a proper amount of diluted dye was dispersed on the board, and the dye was pressed and printed on the product with a rubber roller. The final step was drying. The drying operation was air-drying without special exhaust devices. Dust is an important contaminant that negatively impacts product quality in the printing process, so printing rooms must be dust-free. Air circulation systems in the clean room use inner room air filtering rather than injecting filtered-fresh air from outside. Therefore, there is the possibility of abnormal concentrations of volatile organic compounds in the inner-room circulation system. Moreover, workers of the screen-printing department did not wear personal protective equipment, except surgical masks during work time.

### Work environmental monitoring and measurement

According to the identified material safety data sheets (MSDS), the main raw materials of the dye were barium sulfate, titanium dioxide, and epoxy resin. The solvents mainly consisted of heavy aromatic solvent naphtha, solvent naphtha, BE, and EE (Table 1).

**Table 1:** Summary of MSDS

Product Name	Chemical Name	CAS No	Percent
BG	Ethylene glycol mono butyl ether	111 – 76 - 2	100%
PSR-4000	Titanium dioxide	13463 – 67 - 7	10 – 15%
	Diethylene glycol monobutyl ether acetate	124 – 17 - 4	1 – 5%
	Diethylene glycol monoethyl ether acetate	112 – 15 - 2	1 – 5%
	Epoxy resin and others	Trade secrets	30 – 40%
	Amorphous silica	112945 – 52 - 5	0.3% – 1%
	Barium sulfate	7727 – 43 - 7	40 – 50%
NER-DS	C.I. Pigment blue 15	147 – 14 - 8	< 0.3%
	C.I. Pigment yellow 147	Trade secrets	0.3 – 1%
	Heavy aromatic solvent naphtha	64742 – 94 - 5	10 – 15%
	Solvent naphtha	64742 – 95 - 6	10 – 15%
	Ethylene glycol ethyl ether	110 – 85 - 5	20%
	Modified epoxy acrylate resin and others	Trade secrets	30 – 40%
	Barium sulfate	7727 – 43 - 7	20 – 25%

The work environment measurement is not directly performed, but the most recent measurement results from the date of the workers’ health checkup among the available work environment measurement results.

### Questionnaire study

The self-reporting questionnaire included the following items: name of company, name, history of illness, family history, hepatitis B viral carrier, duration of smoking, amount of tobacco smoked per day, amount of drinking, frequency of drinking in a week, physical activity, and questions about symptoms related to target organs.

### Physical examination and serum biochemistry

The height, weight, and systolic and diastolic blood pressure were measured in all subjects. Fasting blood sugar, total cholesterol, triglycerides, HDL-cholesterol, hemoglobin, hematocrit, complete blood cell counts, and reticulocytes (%) were also measured in all subjects. The body mass index (BMI) was calculated as the weight in kilograms divided by the squared height in meters. In addition, blood examinations, such as tumor marker tests, were only measured in the control group.

### Corrected reticulocyte count

Reticulocyte percentage and corrected reticulocyte count (reticulocyte index, RI) were calculated with the following formula:

Reticulocyte (%) = [Number of Reticulocytes/Number of Red Blood Cells] × 100

Reticulocyte Index (RI) = Reticulocyte (%) × [Patient^’^s Hematocrit/Normal hematocrit]

The maximum reticulocyte percentage considered normal was set as 1.5 for men and 2.0 for women [15]. The RI should be between 1.0% and 2.0% for a healthy individual [15].

### Statistical analyses

Student’s t-test and Pearson’s chi-square test were used to compare differences of hematological parameters between the exposed and control groups. In addition, a multivariate analysis was performed using the multiple logistic regression models to adjust for effective variables that were chosen by the backward elimination methods. The odds ratio (OR) and 95% confidence interval (CI) were calculated for the effect of the exposure on RI. The analysis was performed with the SPSS 18.0 statistical packet (SPSS Inc., Chicago, IL, USA). Results were considered significant when *p* ≤ 0.05.

### Ethics approval and consent to participate

We performed a retrospective chart review and collected no personally identifiable information. The Institutional Review Board of Gachon University Gil Medical Center approved our waiver of written informed consent and review exemption (IRB No. GBIRB2017–112).

## Results

### Work environmental monitoring and measurement

We reviewed most recent measurement results from the date of the workers’ health checkup. Trimethylbenzene, isopropyl alcohol, methyl isobutyl ketone, and toluene were detected at less than 2% of the occupational exposure limit, or were not detectable in the two workplace measurements. Ethylbenzene and xylene were found to have maximum concentrations of less than 5% of the occupational exposure limit in the two measurements of the working environment. EE had the highest concentration at 51.56% of the occupational exposure limit, while BE was measured at 16.75% and cyclohexanone at 14.82% (Table 2).

**Table 2:** Measurements in the work environment

Measured Solvent	OEL (TWA, ppm)	Measurement Second half of 2015 (ppm)		Measurement First half of 2016 (ppm)	
		Primary operator	Secondary operator	Primary operator	Secondary operator
2-Butoxyethanol	20	1.408	2.056	3.350	3.099
2-Ethoxyethanol	5	NE	NE	2.578	2.507
Ethylbenzene	100	3.451	2.904	3.994	4.110
Xylene	100	1.351	1.559	1.815	2.727
Cyclohexanone	25	3.704	2.847	1.507	3.024
Trimethylbenzene	25	0.016	ND	0.327	0.491
Isopropyl alcohol	200	NE	NE	0.484	0.418
Methyl isobutyl ketone	50	ND	ND	NE	NE
Toluene	50	ND	ND	NE	NE

*NE* not measured, *ND* not detectable, *OEL* occupational exposure limit, *TWA* time-weighted average

### General characteristics of the subjects

There were 71 subjects (34 printing workers, 37 office workers), and their characteristics were analyzed using descriptive statistical analysis (Table 3). There were no statistically significant differences in mean age, BMI, and hemoglobin level between the exposed and control groups. The percentage of men in the exposed group was 76.5, which was higher than that of the control group, but no statistically significant difference was found. Additionally, no statistically significant difference was observed between the exposed and control groups in the percentages of workers who smoked and consumed alcohol. No statistically significant difference was found between the two groups in general characteristics.

**Table 3:** General characteristics of exposed group (printing workers) and control group (office workers)

Variables	Exposed group (*n* = 34)		Control group (*n* = 37)		*p*value
	Mean	Standard deviation	Mean	Standard deviation	
Age (years)^a^	31.21	3.88	32.59	6.23	0.355
BMI (Kg/m^2^)^a^	23.51	3.88	23.86	4.49	0.727
Hemoglobin (g/dL)^a^	15.43	1.24	14.87	1.54	0.095
	n	%	n	%	
Sex^b^					0.079
Male	26	76.5%	21	56.8%	
Female	8	23.5%	16	43.2%	
Alcohol consumption^b^					0.124
No	14	41.2%	22	59.5%	
Yes	20	58.8%	15	40.5%	
Smoking^b^					0.702
No	19	55.9%	19	51.4%	
Yes	15	44.1%	18	48.6%	

^a^ *p* values by Student’s t-test

^b^ p values by Pearson’s chi-square test

### Hematologic parameters of the subjects

We compared the mean hematologic parameters of the exposed and control groups (Table 4). No significant differences were found between the exposed group and control group in mean hemoglobin, hematocrit, mean corpuscular volume (MCV), mean corpuscular hemoglobin (MCH), mean corpuscular hemoglobin concentration (MCHC), red blood cell (RBC) and platelet counts, and lymphocyte percentage. However, white blood cell (WBC) counts, reticulocyte counts, and RI were higher in the exposed group than in the control group, and the differences were statistically significant.

**Table 4:** Hematologic parameters of the exposed and control groups

Parameter	Exposed group (*n* = 34)		Control group (*n* = 37)		*p* value
	Mean	Standard deviation	Mean	Standard deviation	
Hemoglobin (g/dL)	15.43	1.24	14.87	1.54	0.095
Hematocrit (%)	45.96	3.19	44.57	4.09	0.117
Red blood cell (10^6^/mm^3^)	5.12	0.54	4.98	0.54	0.264
White blood cell (10^3^/mm^3^)	7.86	2.12	6.36	1.84	0.002^*^
Lymphocyte (%)	36.57	7.52	35.28	9.89	0.541
MCV	88.85	7.99	89.84	4.33	0.513
MCH	29.87	3.07	29.91	1.36	0.946
MCHC	35.57	0.99	33.31	0.83	0.238
Reticulocytes (%)	1.88	0.39	1.60	0.42	0.004^*^
RI (%)	1.92	0.41	1.58	0.48	0.002^*^
Platelet (10^3^/mm^3^)	264.91	51.20	248.41	46.12	0.157

* *p* < 0.05 By Student’s t-test. *MCV* mean corpuscular volume, *MCH* mean corpuscular hemoglobin, *MCHC* mean corpuscular hemoglobin concentration, *RI* reticulocyte index

The percentages of the subjects with hematologic parameters over the reference range between the exposed group and control group were compared using Pearson’s chi-square test (Table 5). No significant differences were found between the exposed group and control group in hemoglobin, hematocrit, RBC count, WBC count, lymphocyte, MCV, MCH, and MCHC. However, reticulocyte counts and RI were higher in the exposed group than in the control group, and the differences were statistically significant. Platelet counts could not be compared since there were no subjects with abnormal counts.

**Table 5:** Hematological abnormalities of exposed and control groups

Parameter		Exposed group *n* (%)	Control group *n* (%)	*p* value
Hemoglobin	Normal	31 (91.2)	35 (94.6)	0.665
	Abnormal	3 (8.8)	2 (5.4)	
Hematocrit	Normal	33 (97.1)	35 (94.6)	1.000
	Abnormal	1 (2.9)	2 (5.4)	
Red blood cell	Normal	23 (67.6)	30 (81.1)	0.194
	Abnormal	11 (32.4)	7 (16.7)	
White blood cell	Normal	27 (79.4)	34 (91.9)	0.178
	Abnormal	7 (20.6)	2 (8.1)	
Lymphocyte	Normal	28 (82.4)	28 (75.7)	0.491
	Abnormal	6 (17.6)	9 (24.3)	
MCV	Normal	28 (82.4)	35 (94.6)	0.103
	Abnormal	6 (17.6)	2 (5.4)	
MCH	Normal	27 (79.4)	34 (91.9)	0.178
	Abnormal	7 (20.6)	3 (8.1)	
MCHC	Normal	33 (97.1)	37 (100)	0.479
	Abnormal	1 (2.9)	0 (0)	
Reticulocyte	Normal	7 (20.6)	24 (64.9)	< 0.001^*^
	Abnormal	27 (79.4)	13 (35.1)	
RI	Normal	22 (64.7)	32 (86.5)	0.032^*^
	Abnormal	12 (35.3)	5 (13.5)	
Platelet	Normal	34 (100)	37 (100)	
	Abnormal	0 (0.0)	0 (0.0)	

* *p* < 0.05 by Pearson’s chi-square test. *MCV* mean corpuscular volume, *MCH* mean corpuscular hemoglobin, *MCHC* mean corpuscular hemoglobin concentration, *RI* reticulocyte index

### Multivariate analysis for reticulocytosis

Reticulocytosis was defined as RI > 2, and a multiple logistic regression was conducted. The variables were chosen by backward elimination methods in the multivariate analysis. In addition, sex variables were included in the final multivariate analysis for adjustment due to the sex ratio difference, even though they had been removed previously by backward elimination. The OR and 95% CI for the exposure were obtained. Age, BMI, drinking, smoking, lymphocyte, platelet count, hemoglobin, MCV, and sex were adjusted in the multiple logistic regression model. The risk of reticulocytosis was 16.3 times (95% CI: 1.53–173.27) higher in the exposure group compared to that in the control group. Moreover, the risk of reticulocytosis decreased by 33% (OR 0.67, 95% CI: 0.49–0.92) when BMI increased by 1 unit. However, other variables, including age, sex, drinking, smoking, lymphocyte, platelet counts, hemoglobin, and MCV, were not significant risk factors for reticulocytosis (Table 6).

**Table 6:** Multivariate analysis using logistic regression^a^

Variable	OR	95% CI
Age	0.92	0.81 – 1.04
Exposure (Office workers vs Printing workers)	16.30*	1.53 – 173.27
Sex (Women vs Men)	3.61	0.10 – 137.24
BMI	0.67*	0.49 – 0.92
Drinking	6.79	0.85 – 54.00
Smoking	0.19	0.03 – 1.38
Lymphocyte	1.09	0.96 – 1.23
Platelet	0.99	0.98 – 1.01
Hemoglobin	0.62	0.20 – 1.91
MCV	1.09	0.93 – 1.27

* *p* < 0.05. BMI, body mass index; *MCV* mean corpuscular volume, *OR* odds ratio, *CI* confidence interval

^a^Adjusted for age, exposure, BMI, drinking, smoking, lymphocyte, platelet count, hemoglobin, MCV, and sex

## Discussion

The objective of this study was to investigate the association between the exposure to EE and BE and reticulocytosis in screen-printing workers. The 34 exposed workers worked at a clean-room isolated space without personal protective equipment, except surgical masks. Due to the recirculating air of the clean-room system, the room is presumed to have constant concentrations of volatile organic compounds and direct contact of dyes and organic compounds with the skin was possible depending on the work type. The main absorption pathway of EE and BE in the exposure group is through inhalation and skin contact. The frequency of skin exposure to dyes and organic compounds was limited, and skin absorption in humans is lower than that of other species (e.g., hairless rat > human) [16]. Thus, inhalation was expected as the main absorption pathway of BE and EE. When humans are exposed to 20 ppm of BE, respiratory uptake is estimated to be ~10.1 μmol/min, which is 57% of the uptake via aspiration [17].

In the identified MSDS and past work environment measurements, ethylbenzene and xylene were the solvents that could have affected the hematopoietic system in addition to BE and EE. Angerer and colleagues reported decreased lymphocytes and hemoglobin levels in workers exposed to mixed solvents, including ethylbenzene, toluene, xylene, ethyl acetate, and butyl acetate [18]. Xylene or other solvents in mixed solvents could have induced the hematopoietic abnormality, and there is no study to corroborate this. For xylene, there is a study of menstrual disorders, especially abnormal bleeding, in female workers exposed to xylene [19]. In our study, however, no female workers in the exposed group checked any symptom of abnormal bleeding on the self-questionnaire, and their hemoglobin levels were normal or beyond the normal limit, which might prove no abnormal bleeding. In addition, ethylbenzene and xylene were measured at less than 5% of the occupational exposure limit in the work environment measurements. Therefore, the hematopoietic effects of exposure to organic solvents other than BE and EE were estimated to be low, although it is difficult to exclude completely the effect of other solvents on hematopoiesis.

As shown on data of measurements in the work environment in Table 2, the concentrations of BE and EE based on the time-weighted average were measured as 51.56% and 16.75% of the occupational exposure limit for each. It is possible that, however, actual exposure levels of BE or EE on exposure group were higher than measurements in the work environment, since companies usually tend to work less during the work environment measurements. Furthermore, BE and EE can be concentrated more as greater workload and longer worktime due to the characteristics of closed work place with inner-room circulation system. Occupational exposure limit were also measured in 8 h based on time-weighted average, so the actual exposed amount of BE and EE were presumed greater if the workers had over-time works as they insisted.

BE and EE absorbed into the body are metabolized to 2-butoxyacetic acid and 2-ethoxyacetic acid, respectively, by the sequential activity of alcohol and aldehyde dehydrogenases, leading to erythrocyte hemolysis [1, 4, 5]. Therefore, the degree of toxicity may be different depending on the relative activities of alcohol and aldehyde dehydrogenases. Generally, metabolism of drugs or carcinogens in humans varies according to genetic polymorphisms affecting transcriptional regulation of the human cytochrome P450 2E1 gene [12]. Many studies are ongoing about general polymorphisms, and several genes and genetic sequences have been identified [20, 21]. Nevertheless, more studies are necessary to confirm the exact mechanisms involved and the effect of genetic differences on individual susceptibility.

Known factors affecting reticulocytosis are age, smoking history, alcohol history, drug history, vitamin deficiency, folate deficiency, menstrual cycle, and other causative diseases (e.g., infection, hematopoietic disease) [22]. Reticulocyte loss occurs with aging. The mean ages between the exposed and control groups were not different statistically. Reticulocyte counts increase with a history of smoking and alcohol use. No statistically significant differences were found in the current drinking and smoking statuses in this study. We were unable to analyze the amount of smoking and drinking in the study, despite their importance, since there was no record of past drinking or tobacco smoked per day for some subjects in the control group. Vitamin B12 or folate deficiency by excessive alcohol intake can induce reticulocytosis due to megaloblastic anemia [23]. Megaloblastic anemia results in MCV elevation, but all subjects were within normal limits, except 8 subjects with lower than normal MCV levels. Therefore, the possibility of reticulocytosis by excessive alcohol intake was estimated to be low. Drug-induced hemolytic anemia also can induce reticulocytosis. High-dose penicillin, quinidine, α-methyldopa, tetracycline, acetaminophen, and ibuprofen are well-known drugs that can induce hemolytic anemia [24]. No medical history or drug history was found in the subjects using the self-reporting questionnaire, except hypertension and diabetes mellitus. Menstrual cycle also might affect the reticulocyte counts for women, but there were no data of menstruation available, so we were unable to confirm the association between menstrual cycle and reticulocyte counts. However, there was no statistical significance in reticulocytosis according to sex differences on the multivariate logistic regression analysis.

In a previous study [11], rats exposed to high concentrations of BE after prior exposure to low concentrations showed greater survival without rapid hematocrit decreases than rats exposed to the high concentrations of BE only. The authors asserted that the red blood cell decreases by low level BE exposure were offset by prompt hematopoiesis, and that the newly formed younger RBCs were resistant to the subsequent lethal dose of BE. In our study, the only hematological parameters that were elevated by EE and BE exposure were reticulocyte percentage and RI. Exposure to BE and EE induces hemolytic episodes at the beginning, but loss of RBC might be offset by prompt hematopoiesis.

In a previous study of rats [7], hematological parameter changes were compared between single exposures to BE or EE and combined exposures to BE and EE. Hematological parameter changes from combined exposures to BE and EE were greater than those of single exposures to BE or EE at the end of the 4-week experiment. For human studies, there are only epidemiologic studies regarding single exposures to BE or EE [4–6]. Additional epidemiologic studies are necessary to compare the hematologic effects of combined exposure and of single exposure.

In the present study, the risk of reticulocytosis decreased with increasing BMI. This is an unexpected result. There has been no study of the association between BMI and reticulocytosis. There are several studies [25–27] about the association between BMI and anemia, but no studies were found to corroborate our results. Further studies are needed to evaluate BMI and reticulocytosis.

There are some limitations of this study. First, it was unable to confirm all chemical compounds potentially affecting hematopoiesis in the exposure group. We could not directly measure the working environment and examine all MSDS for substances used in the workplace because the printing plant had moved abroad at the time of the study. Consequently, there was no way to reaffirm data, and it was impossible to use MSDS directly to confirm all solvent compositions. The dedicated print workers were expected to have the highest solvent exposure, but we could not differentiate the dedicated workers from other process workers. Second, the information of the subjects was limited because the present study was conducted using health examination data. No record was found for overall drug history, infection history, and menstrual cycle. Screen printing work needs venerable skilled workers, so many of them may have a work history in the same field. Consequently, it is necessary to confirm past occupational history as well as current occupational history for the cumulative chemical exposure. However, it was impossible to confirm this information through counseling or additional questionnaires. The exposure group included 10 foreign workers with unidentified nationality, while all subjects were Korean in the control group. Due to race and genetic polymorphisms, it is possible to have different toxic effects of BE and EE, which could affect reticulocytosis. No study was found examining the effects of race on reticulocytes, and we were unable to adjust for race differences. Third, there was sex ratio difference between the exposed group and control group despite of no statistical significance. We tried to correct the sex ratio with stratification analysis, but it could not be performed with a small number of subjects, which might be limitation of this study. Finally, this was a cross-sectional study. Since we did not know basal reticulocyte counts before the workers started at the factory, the temporal relationship was ambiguous, and it was only possible to identify an association between the printing process and reticulocytosis. Although differences were found, it is difficult to distinguish a causal-effect relationship between occupational exposures to BE and EE, and reticulocytosis.

Despite such limitations, the present study has some strengths. First, several previous epidemiological studies have examined the effects of single exposure to either BE or EE on human health. This is, however, the first study to examine the effects of simultaneous exposures to BE and EE on human health. Second, we used special health examination data, and the test items were standardized. Therefore, data on the test items of other print workers exposed to the same hazardous substances can be obtained, and a prospective research design is possible in the future.

## Conclusions

In conclusion, occupational exposure to solvents containing BE and EE was significantly associated with reticulocytosis compared to that of the control group. This study had limitation, however, that not to exclude all other factors affecting reticulocytosis. Therefore, Well-designed, prospective studies need to be conducted to clarify the cause-effect relationship. Additionally, preventive measures should be implemented for workers in the printing process. It is necessary to monitor reticulocytosis persistently with single or combined exposure of BE or EE.

## Abbreviations

AAs: Alkoxyacetic acids
BE: 2-Butoxyethanol
BMI: Body mass index
CI: Confidence interval
EE: 2-Ethoxyethanol
MCH: Mean corpuscular hemoglobin
MCHC: Mean corpuscular hemoglobin concentration
MCV: Mean corpuscular volume
MSDS: Material safety data sheet
OR: Odds ratio
RBC: Red blood cell
RI: Reticulocyte index
WBC: White blood cell
